# The Fate of the Agitator

**Published:** 1918-08-10

**Authors:** 


### SILENCE OF THE NURSE.

### The Fate of the Agitator.

A friend of mine who was present at a recent meeting of a certain hospital board told me of what took place. An application was submitted from two or three nurses asking for an increase of pay. The matron added her comments for the consideration of the board, and pointed out that one of the applicants was "an agitator," and was really responsible. She recommended that no increase be awarded, and added that if the agitating person left she would not regret her departure. The committee immediately and without discussion passed a resolution that in their opinion the salaries paid were adequate, and expressed regret that the application had to be refused. When I heard the story I immediately thought of Lord Knutsford and his experience of a growing tendency to growl. I happen to know this particular hospital. The matron is an excellent lady in every respect, and I am sure she felt that she was doing her duty. The point of the story is that the supreme duty of the hospital matron is to her board, and that in performing this she finds it natural and effectual to crack the whip. I made particular inquiry as to whether the justice or otherwise of the application was discussed or analysed. Not a word. All that I could gather is that one of the staff is marked down as an undesirable because she growled, and apparently induced others to growl in unison. History does not relate how or in what terms the matron conveyed the board's message to the delinquents. History, in all probability, never will record the fate of the "agitator," but it is not unreasonable to suppose that at the earliest opportunity she may receive a polite hint that the board do not appreciate her services. She may be an excellent nurse, but she is an "agitator." She is an articulate worker, and this is the manner of her relegation to the regions of silence.

### Too Pessimistic a View.

I confess that I am not optimistic as to any change of procedure being effected. So long as the problem of finding the money is paramount there must of necessity be a sort of Treasury Remembrancer whose office is to cut down expenditure to the irreducible minimum. This is done by forcing the staff to work for long hours, by imposing on probationers a longer term than three years' service before awarding their diploma, and by driving agitation for improvement under the surface. And I cannot conceive of the average matron ever appearing before her board as the champion of liberality, even if she were convinced that justice was not being done. [*Note.—In fact some of the best matrons have not infrequently done so.*—Ed. The Hospital.]

### The Cream of the Profession.

This subject is, as I have already stated, distinctly delicate. No Nurses' Committee, including the Executive Committee of the College of Nursing, and including any Committees established under the auspices of the opponents of the College, has ever been formed that did not consist in preponderating degree of hospital matrons. No Nurses' Committee is likely ever to be otherwise formed. I might go further, and say that it is impossible, even if it were desired, to ignore the matron. In my opinion this is neither desired nor desirable. She is the cream of the profession. By her intrinsic merit she has risen, and the most radical and revolutionary sister who becomes matron must perforce wear the mantle of her Order. I have thought the subject out, and I invite others to do the same. A hospital matron who is not an economist is unfit for her task. Nevertheless, if the matter is delicate it is also of supreme importance, and nothing but stalemate is to ensue if the obstacles to progress are not surmounted. What I see ahead is this: that the College of Nursing is faced with a problem of considerable dimensions, one that cannot be solved save by statesmanship of a high order. The grievances of the nurse are economic, and their effectual redress means expenditure.

### An Example for the College to Follow.

The Executive of the College consists almost exclusively of the matrons of hospitals, whose major functions day by day consist of guarding against unnecessary expense, and I ask is it possible for them to take an unbiassed view of the economic claims of the staff? I do not think it is. Let me illustrate. The Civil Service of the kingdom recently clamoured for a war bonus. They were not inarticulate. They agitated and pressed their claims on Parliament, with the result that an independent Conciliation Board with plenary powers was appointed. Now the British Treasury corresponds with the hospital matron, and when Treasury representatives appeared before the Conciliation Board they replied in the negative. It was their professional and everlasting No. But the articulate Civil Servant triumphed, and the award of the Conciliation Board was not illiberal. Neither was it liberal; but it was just, and I do not think that British nurses want more than justice.

### A Plan of Campaign Suggested.

May I suggest for the consideration of the College of Nursing a plan of campaign? I do so with diffidence and hesitation, for in the first place I have not been asked, and in the second I realise the difficulties that bristle in the way. Nevertheless, I also realise that the nursing press is the best friend of the College, and at the same time the press is the only means which the nurse possesses for expressing her views and her ambitions. The professed desire of the College is the betterment of the economic condition of the profession, and already, at this early stage of its existence, ten thousand working nurses, members of the College, await a programme of propaganda. My suggestion, which I hope in the future to elaborate, is that the Executive of the College should appoint a Board of Conciliation corresponding with that appointed by Parliament to settle the claims of the Civil Service, this Board to be exempt from hospital matrons as the Government Conciliation Board is exempt from Treasury officials. Let such a sub-committee of the College be persons free from bias of any kind, and let them take all the circumstances into consideration and draw up a Hospital Nurses' Charter of Employment which the Executive can adopt as its own and press on hospital boards for adoption.

### Immediate Action is Pressing.

The need for high statesmanship and immediate action is pressing. The war is the nurse's opportunity for emancipation. She has served the nation and the Empire as patriotically and as nobly as the Navy and the Army, and the nation appreciates her sacrifice. The wisest of us know not what the morrow may bring forth.

IERNE.

